# Supplementary material for: Identification of photoperiod-induced specific miRNAs in the adrenal glands of Sunite sheep (Ovis aries)
Source: Front Vet Sci. 2022 Jul 22;9:888207. doi: 10.3389/fvets.2022.888207 (PMC9354845; doi:10.3389/fvets.2022.888207)
Supplement: Supplementary file 1 [file Data_Sheet_1.ZIP › Supplementary file/miRDeep2 file.zip!/miRDeep2 file/Know_Structure/SPLP42A2/oar-mir-544.pdf]

miRBase precursor : oar-mir-544
Total read count : 479
oar-miR-544-5p read count : 445
oar-miR-544-3p read count : 28
remaining reads : 6

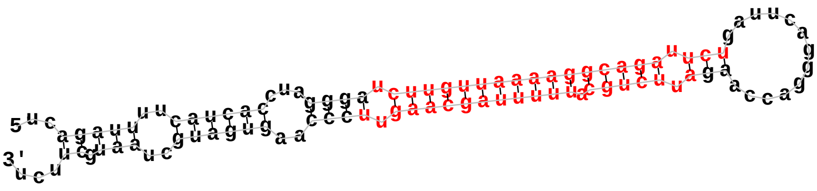

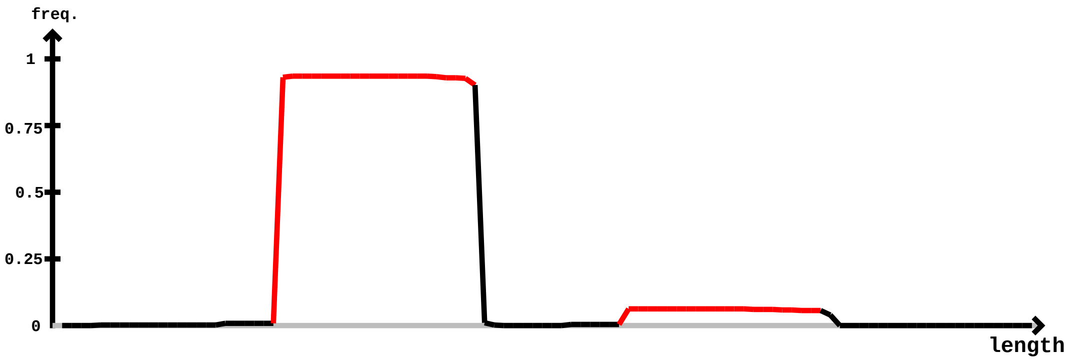

oar-miR-544-5p

oar-miR-544-3p

```
5'- ucagauuuucaucaccuagggaucuuguuaaaaggcagauucugauucagggaccaagauucugcauuuuuagcaaguucccaagugaugcuaaugcuucu   -3'    exp
    ..(((((..((((((...((((.((((((((((((((((.(((.............))).))))).))))))))))).)))).))))))..))).))...    reads       mm          sample
    ...gauuuucaucaccuaggga...............................................................................    1           0           seq
    ................uagggaucuuguuaaaaggcag...............................................................    1           0           seq
    ................uagggaucuuguuaaaaggcaga..............................................................    2           0           seq
    ......................ucuuguuaaaaggcagauu............................................................    1           0           seq
    ......................ucuuguuaaaaggcagauuc...........................................................    11          0           seq
    ......................ucuuguuaGaaggcagauuc...........................................................    1           1           seq
    ......................ucuuCuuaaaaggcagauucu..........................................................    1           1           seq
    ......................Ccuuguuaaaaggcagauucu..........................................................    4           1           seq
    ......................ucuuAuuaaaaggcagauucu..........................................................    1           1           seq
    ......................ucuuguuaaaaggcagauucA..........................................................    15          1           seq
    ......................ucuuguuaaaaggcagGuucu..........................................................    2           1           seq
    ......................ucuuguuaaaaggcagauuUu..........................................................    1           1           seq
    ......................ucuuguuaaaaggUagauucu..........................................................    1           1           seq
    ......................ucuuguGaaaaggcagauucu..........................................................    1           1           seq
    ......................ucuuguuaCaaggcagauucu..........................................................    1           1           seq
    ......................ucuuguuaaaaAgcagauucu..........................................................    1           1           seq
    ......................ucuuguuaaaaggcagauucu..........................................................    397         0           seq
    ......................ucuuguuaaaaggcagauucuA.........................................................    1           1           seq
    ......................ucuuguuaaaaggcagauucuU.........................................................    1           1           seq
    ......................ucuuguuaaaaggcagauucug........................................................    2           0           seq
    ......................ucuuguuaaaaggcagauucuUa........................................................    1           1           seq
    .......................cuuguuaaaaggcagauucu..........................................................    2           0           seq
    ....................................................accaagauucugcauuuuu..............................    1           0           seq
    ....................................................accaagauucugcauuuuuagc...........................    1           0           seq
    ..........................................................auucugcauuuuuagcaa.........................    1           0           seq
    ..........................................................auucugcauuuuuagcaaguu......................    8           0           seq
    ..........................................................auucugcauuuuuagcaaguuc.....................    13          0           seq
    ..........................................................auucugcauuuuuagcaaguuU.....................    6           1           seq
```
